# Supplementary material for: Computational analysis of pathological images enables a better diagnosis of TFE3 Xp11.2 translocation renal cell carcinoma
Source: Nat Commun. 2020 Apr 14;11:1778. doi: 10.1038/s41467-020-15671-5 (PMC7156652; doi:10.1038/s41467-020-15671-5)
Supplement: Supplementary file 1 — Supplementary Information [file 41467_2020_15671_MOESM1_ESM.pdf]

Supplementary Information

Computational analysis of pathological images enables a better diagnosis of TFE3 Xp11.2 translocation renal cell carcinoma

Cheng et al.

Supplementary Figures: 1 - 4

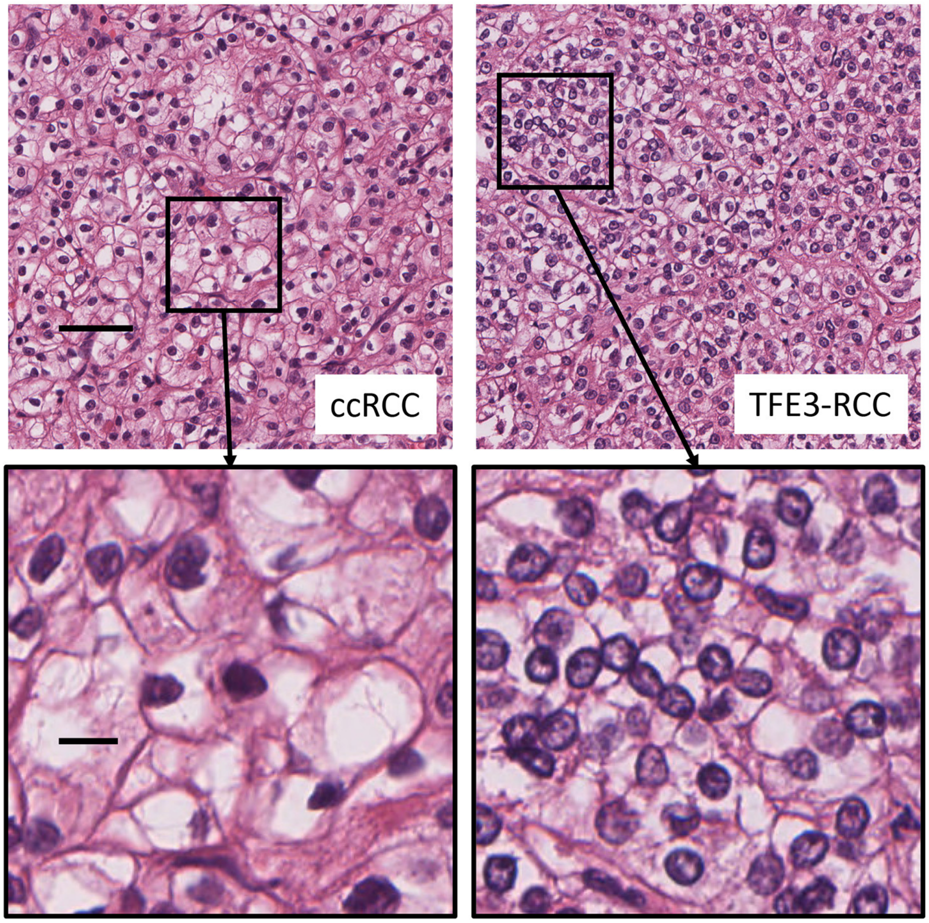

Supplementary Fig. 1 Image patches illustrating more cell clumps in TFE3-RCC than in ccRCC. This can be inferred by our cell density related image features such as distMean_bin1 and distMean_bin2. Scale bars: 50 µm (first row) and 10 µm (second row).

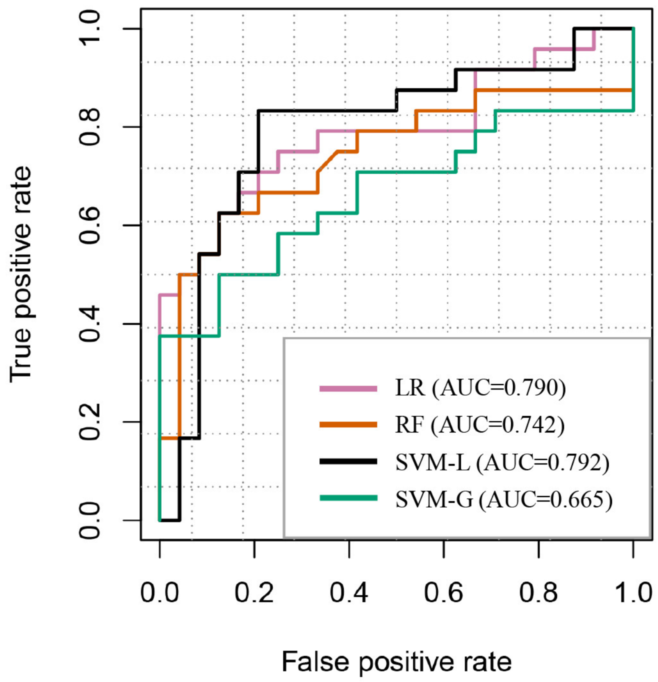

Supplementary Fig. 2 ROC curves for classifying TFE3-RCC and ccRCC. Models were trained using dataset 1 and evaluated using dataset 2 without transforming the color appearance of the slides in dataset 2 into that in dataset 1. The 95% confidence intervals for the AUCs: LR (0.657-0.923), RF (0.591-0.894), SVM-L (0.652-0.931), and SVM-G (0.501-0.829). LR, logistic regression; RF, random forest; SVM-L, SVM with linear kernel; SVM-G, SVM with Gaussian kernel.

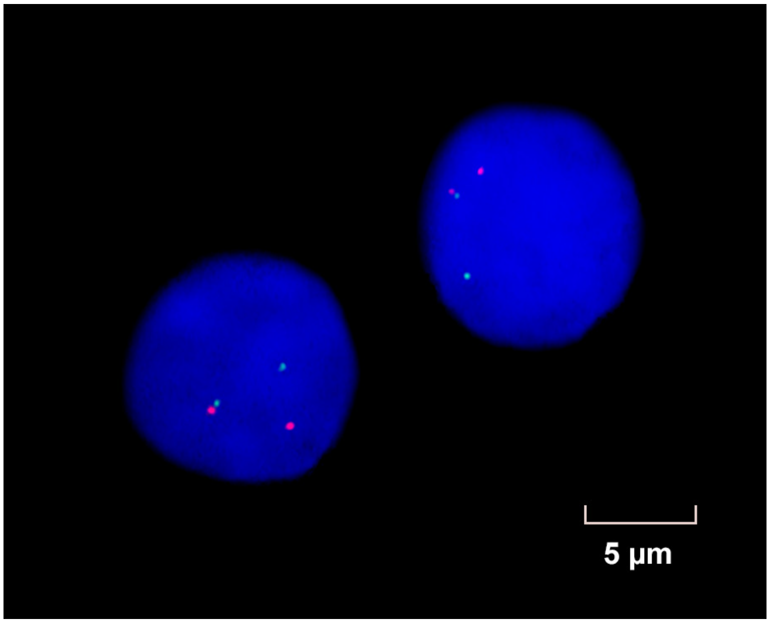

Supplementary Fig. 3 A positive result of the TFE3 break-apart FISH assay. The TFE3 fusion results in a split-signal pattern.

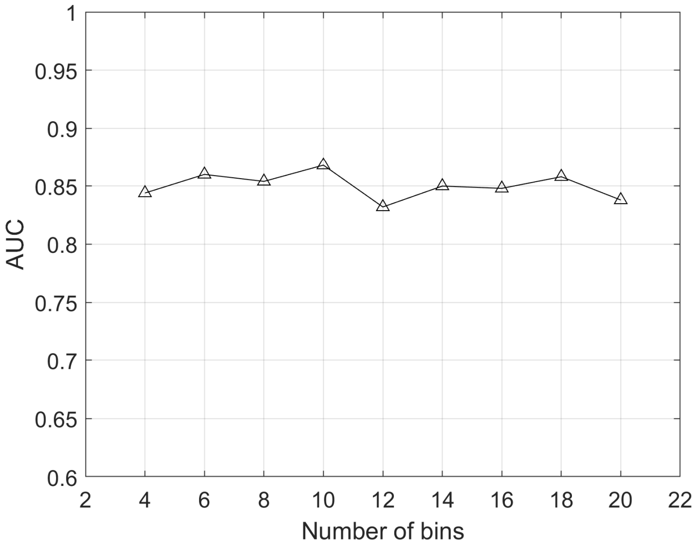

Supplementary Fig. 4 Plot of AUC vs number of bins. A five-fold cross-validation (CV) approach is used to choose the number of bins on dataset 1. The vertical axis represents the average AUC of five-fold CV using a logistic regression classifier for each of the numbers ranging from 4 to 20. The classification performance is robust in this bin size range, and this number is set to 10 in our study.
